# Burden of *Giardia duodenalis* Infection and Its Adverse Effects on Growth of Schoolchildren in Rural Malaysia

**DOI:** 10.1371/journal.pntd.0002516

**Published:** 2013-10-31

**Authors:** Hesham M. Al-Mekhlafi, Mohamed T. Al-Maktari, Rohana Jani, Abdulhamid Ahmed, Tengku Shahrul Anuar, Norhayati Moktar, Mohammed A. K. Mahdy, Yvonne A. L. Lim, Rohela Mahmud, Johari Surin

**Affiliations:** 1 Department of Parasitology, Faculty of Medicine, University of Malaya, Kuala Lumpur, Malaysia; 2 Department of Medical Parasitology, Faculty of Medicine, Sana'a University, Sana'a, Yemen; 3 Department of Applied Statistics, Faculty of Economics and Administration, University of Malaya, Kuala Lumpur, Malaysia; 4 Department of Biology, Faculty of Natural and Applied Sciences, Umaru Musa Yar'adua University, Katsina. Katsina State, Nigeria; 5 Department of Medical Laboratory Technology, Faculty of Health Sciences, Universiti Teknologi MARA (Puncak Alam Campus), Selangor, Malaysia; 6 Department of Parasitology and Medical Entomology, Faculty of Medicine, Universiti Kebangsaan Malaysia, Jalan Raja Muda Abdul Aziz, Kuala Lumpur, Malaysia; Georgetown University, United States of America

## Abstract

**Background:**

*Giardia duodenalis* infection and malnutrition are still considered as public health problems in many developing countries especially among children in rural communities. This study was carried out among Aboriginal (Orang Asli) primary schoolchildren in rural peninsular Malaysia to investigate the burden and the effects of Giardia infection on growth (weight and height) of the children.

**Methods/Findings:**

Weight and height of 374 children aged 7–12 years were assessed before and after treatment of *Giardia* infection. The children were screened for *Giardia* parasite using trichrome staining technique. Demographic and socioeconomic data were collected via face-to-face interviews using a pre-tested questionnaire. Overall, 22.2% (83/374) of the children were found to be infected with *Giardia*. Nutritional status of children was assessed and the results showed that the mean weight and height were 23.9 kg (95% CI = 23.3, 24.5) and 126.6 cm (95% CI = 125.6, 127.5), respectively. Overall, the prevalence of severe underweight, stunting and wasting were 28.3%, 23.8% and 21.0%, respectively. Multiple linear regression analyses showed sex, *Giardia* infection and household monthly income as the significant determinants of weight while sex and level of mother's education were the significant determinants of height. Weight and height were assessed at 3 and 6 months after treatment of *Giardia* infection. It was found that *Giardia* infection has a significant association with the weight of children but not with height.

**Conclusions/Significance:**

This study reveals high prevalence of *Giardia* infection and malnutrition among Aboriginal children in rural Malaysia and clearly highlights an urgent need to identify integrated measures to control these health problems in the rural communities. Essentially, proper attention should be given to the control of *Giardia* infection in Aboriginal communities as this constitutes one of the strategies to improve the nutritional status of Aboriginal children.

## Author Summary

*Giardia* infection, a neglected infection caused by the protozoan parasite *Giardia duodenalis* is prevalent worldwide especially among young children in rural areas of the tropics and subtropics. In Malaysia, *Giardia* infection and protein-energy malnutrition coexist in Aboriginal (Orang Asli) communities with high prevalence among school-aged children. We screened 374 schoolchildren in Lipis and Raub districts, Pahang, Malaysia for the presence of *Giardia* infection and investigated the effects of this infection on the growth of children. Overall, 22.2% of the children studied were infected with *Giardia*. Nutritional status of children was assessed and we found that the prevalence of severe underweight, stunting and wasting were 28.3%, 23.8% and 21.0%, respectively. *Giardia* infection was identified as a significant determinant of weight among these children. Moreover, it was found that *Giardia* infection has a significant association with the weight of children but not with height. The control of *Giardia* infection should be given prominence as it is one of the strategies to enhance the nutritional status of Aboriginal children.

## Introduction

*Giardia duodenalis* (syn. *G. intestinalis*; *G. lamblia*) is the most frequently reported intestinal parasite worldwide, especially among children in developing countries, with a prevalence rate of 10–50% [1]. It has also been identified as a main cause of diarrhoea among young children in day care centers and among travellers from developed countries [2], [3]. Waterborne and foodborne transmission is the most frequent mode of spread besides person-to-person transmission [4], [5]. *Giardia* infection may cause acute or chronic diarrhea or be present as an asymptomatic infection [6]. Often, patients suffering from acute infection present with diarrhoea, abdominal pain and the clinical manifestations of malabsorption [7]. Chronic infection is usually associated with clinical manifestations of malnutrition and micronutrient deficiencies, especially vitamin A deficiency (VAD) and iron deficiency anaemia (IDA) [8], [9]. A significant association between the chronic infection and poor cognitive function has also been reported among children [6].

Protein-energy malnutrition (PEM) is probably the world's major public health problem especially in Africa and Southern Asia where about 70% of all the children are malnourished [10]. Despite the reduction in the global prevalence of stunting and underweight from 40% and 25% in 1990 to 26% and 16%, respectively in 2011, the target of a 40% reduction by 2025 in the global number of children under-five years of age who are stunted could not be reached under the current rates of decline [11]. Malnutrition and parasitic infections coexist in poor socioeconomic communities of the developing countries [12], [13]. The association of *Giardia* infection with malnutrition has been investigated throughout many studies in different countries and the studies have yielded a variety of results. Whilst some studies showed negative impacts of the infections on the nutritional status of children [14]–[17], other studies found no association [18]–[20].

In Malaysia, although there is increasing concern about the burden of these problems in rural areas, data on the association of *Giardia* infection with malnutrition are largely lacking. Previous cross-sectional studies among Aboriginal children in peripheral and rural areas of Selangor and Pahang identified *Giardia* infection as a significant risk factor of malnutrition and poor vitamin A status [9], [21]. However, these studies were based on a single point data analysis (cross-sectional). Within this context, the aim of the present study was to investigate the burden of *G. duodenalis* infection and its effect on the growth (weight and height) of Aboriginal primary school-age children in rural Malaysia.

## Materials and Methods

### Ethical statement

The protocol of the study was approved by the Medical Ethics Committee of the University of Malaya Medical Centre, Malaysia (Reference Number: 788.74). Before the commencement of the present study, meetings were held with the headmaster, teachers, heads of the villages, parents, and the schoolchildren. The purpose, procedures, potential risks and benefits of the study were explained to the parents and children. During the meeting, they were also informed that their participation was totally voluntarily and they could withdraw from the study at any time without citing reasons for doing so. Written and signed or thumb-printed informed consents were taken from parents or guardians on behalf of their children, and these procedures were approved by the Medical Ethics Committee of the University of Malaya Medical Centre. All the infected children were treated with a 3-day course of 400 mg albendazole tablets. Albendazole is also considered as the drug of choice for *Ascaris*, *Trichuris* and hookworm infections. By the end of 6 months assessment, drugs were distributed to all children who were found to be infected.

### Study area

**Figure 1:**
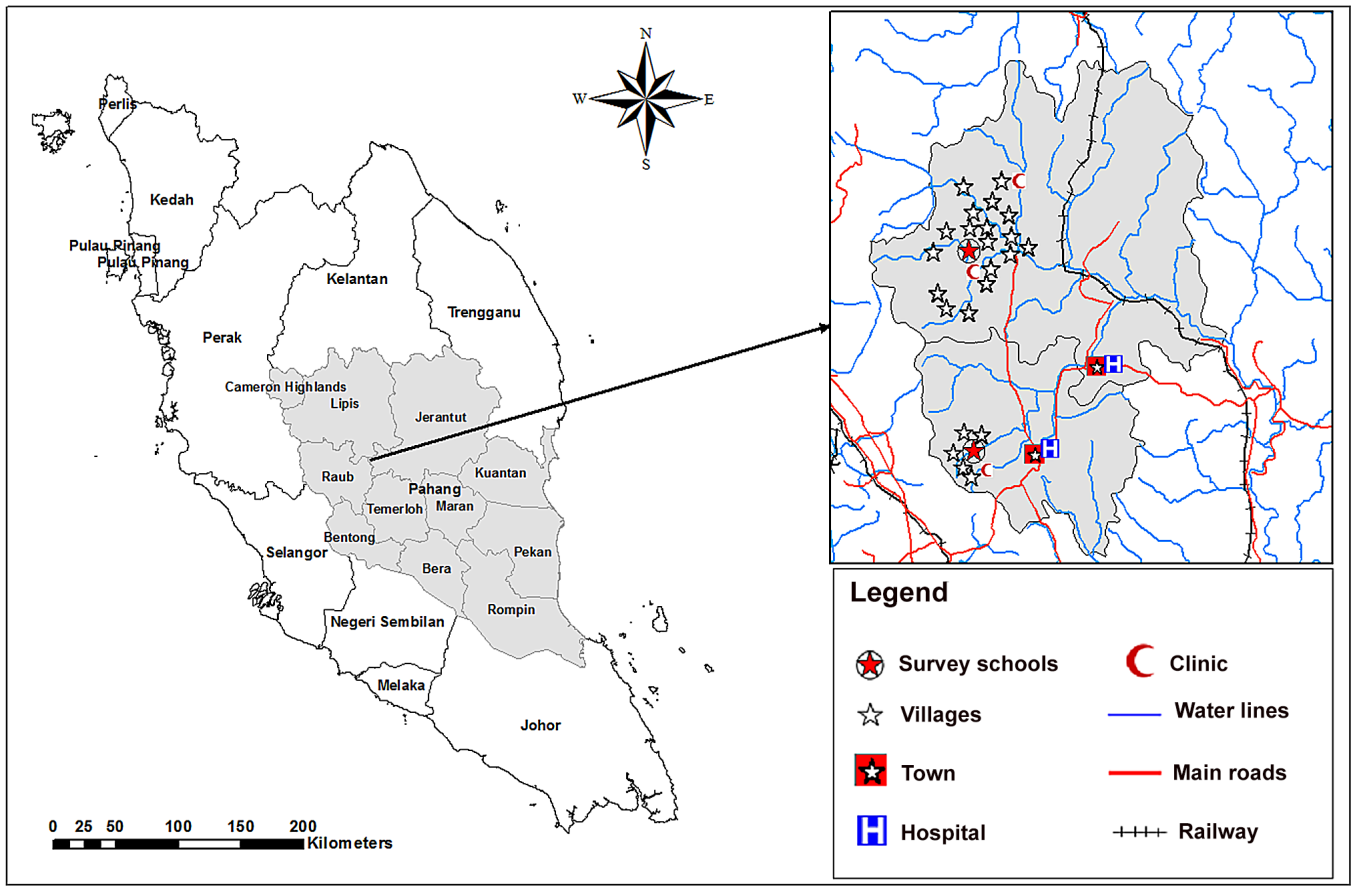
A geographic map showing Pahang state and the location of the selected schools and villages in Lipis and Raub districts.

A longitudinal study with before and after treatment follow up assessments was carried out in primary schools for Aboriginal children in Raub and Lipis districts, Pahang state, Malaysia. Data collection was carried out between March and October 2010. Sekolah Kebangsaan Satak (National School of Satak) in Raub and Sekolah Kebangsaan Betau (National School of Betau) in Lipis were selected for this study (Figure 1). The study area in Raub district, located about 140 km northeast of Kuala Lumpur, has five main Aboriginal settlements namely; Satak, Rensong, Ruai Hulu, Ruai Hilir, and Kelang. The study area in Lipis district, located about 200 km northeast of Kuala Lumpur, has 18 villages. Adequate sanitation facilities is the main predictor for acquiring intestinal parasitic infections, especially *Giardia* and soil-transmitted helminthes, in the Aboriginal and rural communities. The food supply in these communities is constantly poor in energy and periodically low in protein [22], [23]. Most of these impoverished people are subsistence farmers, completely dependent on their environment for survival.

### Study population

**Figure 2:**
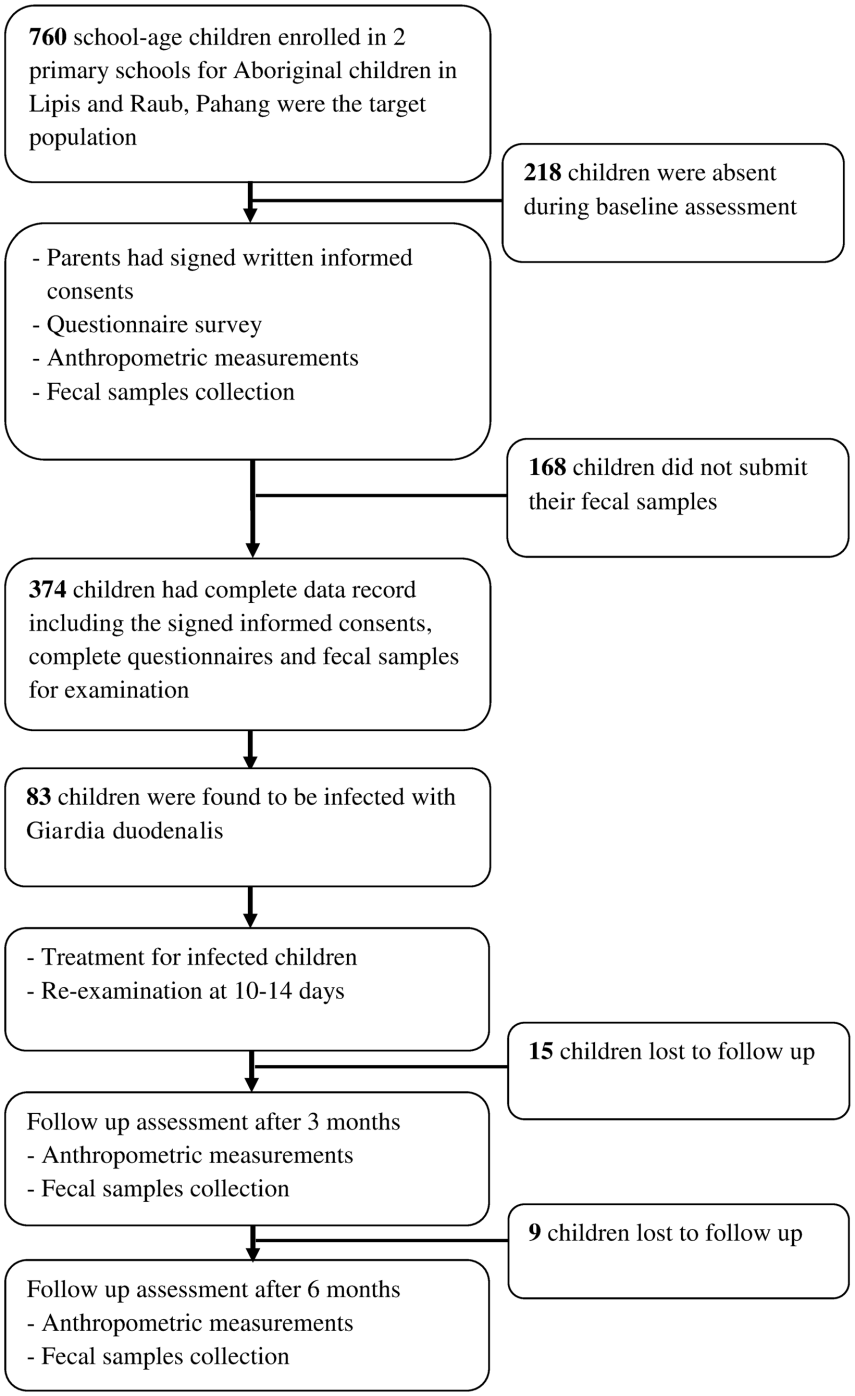
Flow chart of the participation and compliance in the present study.

The schools had a total enrolment of 760 children in grades one to six. However, 218 students were absent during the enrolment day. All attending children were invited to participate in the study and those who agreed were enrolled. Out of the 542 students, 374 eligible children, aged 7–12 years, agreed to participate in this study and had delivered fecal samples for examination. Subsequently, 15 and 9 children were lost to follow up at 3 and 6 months assessments, respectively (Figure 2). For ethical reasons, a placebo control group was not included.

### Questionnaire

A structured questionnaire was prepared in English and then translated to Bahasa Malaysia (the national language for Malaysia). The questionnaire was designed to collect information on the demographic, socioeconomic and environmental background, personal hygiene and practices and health status of the participants. For this, the parents were interviewed in their home settings by two field assistants, one from the school and the other from the Department of Parasitology, University of Malaya. Both assistants were trained by the principal investigator on the purpose of the study and on how to administer the questionnaire.

### Anthropometric measurements

All children underwent anthropometric measurements for weight and height according to this procedure: children were weighed wearing the school uniforms (empty pockets, without belts and barefooted) using a calibrated 0.1 kg intervals SECA scale (709-USA). Using the same instrument which has a scaled sliding head piece, height of children was measured to the nearest millimeter. For quality control, the scale was calibrated regularly and measurements were taken twice by different persons and the mean value was recorded. To assess the nutritional status of children, weight-for-age Z-score (WAZ), height-for-age Z-score (HAZ) and weight-for-height Z-score (WHZ) were calculated and used as indicators for underweight (an overall indicator for malnutrition), stunting or shortness (chronic malnutrition) and wasting or thinness (acute malnutrition), respectively. These Z-scores were derived from the measurements of weight and height using EpiNut Anthropometry in Epi Info program.

The Z-scores were evaluated according to the median values of the National Center for Health Statistics (NCHS), which are based on those recommended by the World Health Organization. Using these reference values, children who had Z-score below −2 standard deviations of the NCHS median values were considered to have severe malnutrition and Z-scores between −1 and −2 standard deviations were considered to have moderate malnutrition [24].

### Fecal sample examination

Fresh fecal samples were collected into clean 100 mL wide-mouth screw cap containers. The participants were instructed to scoop a suitable amount of fecal sample, using a provided scoop into the container. Then, the containers were placed into zip-locked plastic bags and transported for examination at the stool processing laboratory in the Department of Parasitology, University of Malaya. Approximately 10 g of each fecal sample was kept in Poly Vinyl Alcohol (PVA). Detection of *G. duodenalis* was performed using trichrome staining technique [25]. *G. duodenalis* infection was recorded as positive when cysts and/or trophozoites were detected in the stained fecal smear. Negative fecal samples were re-examined by formalin ether sedimentation technique as described by Cheesbrough [26] before the negative results were confirmed. The unpreserved fecal samples were examined using the Kato-Katz and Harada-Mori fecal cultivation techniques for the presence of soil-transmitted helminths: *Ascaris lumbricoides*, *Trichuris trichiura* and hookworm eggs [27], [28]. To determine the worm burden, egg counts were taken and recorded as eggs per gram of feces (epg) for each positive sample and the intensity of infections was graded as heavy, moderate or light according to the criteria proposed by the WHO [29].

Fecal samples were collected and examined by the same methods at 14 days, 3 and 6 months after the administration of treatment for the infected children. For quality control, duplicate analysis was performed on 93 (25%) randomly collected samples. Moreover, the samples were coded only with numbers and the technicians at the diagnostic laboratory were blinded to the code.

### Treatment

After the baseline assessment for the presence of *Giardia* infections, all the infected children were treated with 400 mg albendazole tablets. Penggabean et al. have evaluated the efficacy of albendazole in treating *Giardia* infection among 917 subjects in rural Malaysia and found that a 3-day course of 400 mg albendazole is highly effective against *Giardia* with a cure rate of 96.6% [30]. Similar findings were reported in a remote Aboriginal community in Australia and in Bangladesh [31], [32]. Moreover, a previous meta-analysis on the effectiveness of albendazole compared with metronidazole concluded that albendazole could be used as an alternative and/or a replacement for the metronidazole in the treatment of *Giardia* infection [33].

The tablets were available in small packets of two tablets; each white chewable tablet contains 200 mg albendazole as the active ingredient. The orange flavor encouraged the children to chew the tablets before swallowing. Each child was given two tablets daily for 3 days. A researcher and a medical officer supervised the treatment and asked each child to open their mouth to confirm the tablets have been swallowed. Fecal samples were collected on the 10–14 days post treatment and examined to ascertain the effectiveness of the treatment. All children who received the treatment were reported free from *Giardia* parasites.

### Statistical analysis

Data was double-entered by two different researchers into Microsoft Office Excel 2007 spreadsheets. A third researcher cross-checked the two data sets for accuracy and created a single data set. Data analysis was performed by using *Statistical Package for Social Sciences for Windows* (SPSS) version 13. Demographic, socioeconomic, environmental and behavioural characteristics were treated as categorical variables and presented as frequencies and percentages. All continuous variables were evaluated for normality by Kolmogorov-Smirnov Z test and means and 95% confidence intervals (95% CI) were calculated. For inferential statistics, the dependent variables were weight and height while the independent variables were *Giardia* infection (main factor), demographic factors (age and gender) and socioeconomic factors (parents' educational levels, parents' employment status, household monthly income, family size). To investigate the impact of *Giardia* infection on growth, independent t-test was used to examine the differences in weight and height between the infected and non-infected children. The changes in these parameters between the baseline and the follow up assessment were examined by paired t-test. These comparisons were adjusted for age and sex. Chi-square test was used to examine the significance of the associations and differences in frequency distribution of variables. A repeated-measures ANOVA on the means of weight and height was used to investigate the trend of incremental change over time by *Giardia* infection status. Wilks' Lambda statistics and multivariate eta squared were reported to demonstrate the effect size in ANOVA tests.

Multiple linear regression analyses were used to identify the determinants of weight and height. Beta (β) coefficient and its standard error and 95% CI were reported for the significant determinants. All variables that showed significant difference with *P*≤0.25 in the univariate analysis were used to develop the multiple linear regression models as suggested by Bendel and Afifi [34]. All tests were considered significant at *P*<0.05.

## Results

### General characteristics of the study population

Three hundred and seventy four children aged 7–12 years with a mean age of 9.4 years (95% CI = 9.2, 9.6) participated in this study. School absenteeism and dropout rates in these communities were found to be high and this could be attributed to poverty, in general, as children of poor families being forced to work or to help their parents in their work and daily activities. Moreover, other factors such as diseases or the lack of interest in gaining an education could also be associated with school dropout and absenteeism among these children. The general characteristics of the subjects and their families are shown in Table 1. Approximately half of the fathers and mothers had no formal education and about two-thirds (63.4%) of the families had low household monthly income (<RM500; US$1 = MYR3.05). Only one-third and one-fifth of the fathers and mothers were working, respectively, as laborers in the palm oil or rubber plantations. Most of the houses were made of woods or bamboo and about half of the houses had piped water supply (gravity-fed) and electricity. The socioeconomic characteristics were found to be homogeneous in these two different Aboriginal communities.

**Table 1:** General baseline characteristics of Aboriginal schoolchildren who participated in this study (n = 374).

Characteristics	Frequency (%)
Age groups	
≥10 years	129 (34.5)
<10 years	245 (65.5)
Gender	
Females	198 (52.9)
Males	176 (47.1)
Socioeconomic status	
Fathers' education level (at least 6 years)	191 (51.1)
Mothers' education level (at least 6 years)	175 (46.8)
Low household income (<RM500)	237 (63.4)
Working fathers	114 (30.5)
Working mothers	80 (21.4)
Large family size (>7 members)	80 (21.4)
Piped water supply	194 (51.9)
Electricity	200 (53.5)
Presence of toilet in house	176 (47.1)

### Parasitology

The prevalence and distribution of *Giardia* infection according to age and gender are shown in Table 2. Of the 374 participants, 83 (22.2%) were found to be positive for *Giardia* infection. Overall, the prevalence of *Giardia* infection was higher among children aged <10 years compared to those aged ≥10 years (27.4% vs 16.0%), however, the difference was not statistically significant (χ^2^ = 2.171; *P* = 0.141). Similarly, there was no significant difference in the prevalence of *Giardia* between males and females (24.6% vs 20.0%; χ^2^ = 1.134; *P* = 0.287). *Trichuris trichiura*, *Ascaris lumbricoides*, hookworm infections and *Entamoeba histolytica/dispar* were detected in 71.6%, 40.2%, 10.1% and 9.2% of the samples, respectively. Regarding co-infections, about 60% of the children had *Giardia* with *Ascaris* and/or *Trichuris*. Fecal samples were collected and examined at 3 and 6 months and infected child were treated. There were 1 and 2 cases of *Giardia* infection at 3 and 6 months among those reported negative at baseline. On the other hand, there were 3 and 5 cases of reinfection at 3 and 6 months among those reported positive at baseline.

**Table 2:** Prevalence of *Giardia* infection among Aboriginal primary schoolchildren in Satak, Raub, Pahang according to age and gender (n = 374).

Age/Gender	*Giardia* infection		
	No. examined	No. infected	Prevalence (%)
Age group (years)			
≥10	129	23	17.8
<10	245	60	24.5
Gender			
Male	179	44	24.6
Female	195	39	20.0
Total	374	83	22.2

### Nutritional status

Results of the weight, height and the prevalence of malnutrition (underweight, stunting and wasting) are presented in Table 3. The mean weight of these participants was 23.9 kg (95% CI = 23.3, 24.5) and their mean height was 126.6 cm (95% CI = 125.6, 127.7). The mean height of females was significantly higher than males (127.5 cm; 95% CI = 126.7, 129.0 vs 125.6 cm; 95% CI = 124.2, 126.6; t = 1.985; *P* = 0.048). However, there was no significant difference in the mean weight between female and male participants (24.3 kg; 95% CI = 23.3, 25.3 vs 23.5 kg; 95% CI = 22.7, 24.2; t = 1.300; *P* = 0.188). The overall prevalence of severe underweight, severe stunting and severe wasting were 28.3%, 23.8% and 21.0%, respectively.

**Table 3:** Prevalence of malnutrition at baseline among Aboriginal primary schoolchildren in Satak, Raub, Pahang (n = 374).

Age/Gender	Weight (kg)*	Height (cm)*	Criteria					
			Underweight		Stunting		Wasting	
			Normal/Mild n (%)	Severe n (%)	Normal/Mild n (%)	Severe n (%)	Normal/Mild n (%)	Severe n (%)
Age group (years)								
≥10	29.4(28.4, 30.4)	134.9(133.7, 136.0)	85(65.9)	44(34.1)	62(48.1)	67(51.9)	41(82.0)	9(18.0)
<10	21.0(20.5, 21.5)	122.2(121.2, 123.1)	183(75.0)	62(25.3)	27(11.0)	218(89.0)	192(78.4)	53(21.6)
Gender								
Male	23.5(22.7, 24.2)	125.6(124.2, 126.6)	114(65.1)	62(35.2)	115(63.3)	61(34.7)	119(85.6)	20(14.4)
Female	24.3(23.3, 25.3)	127.5(126.7, 129.0)	154(77.8)	44(22.2)	170(85.9)	14(14.1)	114(73.1)	42(26.9)
Total	23.9(23.3, 24.5)	126.6(125.6, 127.7)	268(71.8)	106(28.3)	285(76.2)	89(23.8)	233(79.0)	62(21.0)

n represents the number of subjects.

* Values are mean (95% confidence interval).

### Determinants of nutritional status (weight and height)

Differences in mean weight and height of participants according to independent variables were examined by independent samples t-test and the results are shown in Table 4. Besides the biological effect of age and gender, the mean weight was found to be significantly lower among children of mothers with <6 years of education (22.9 kg; 95% CI = 22.1, 23.7; t = 3.336; *P* = 0.001), those from families with low household monthly income (<RM500) (22.9 kg; 95% CI = 22.2, 23.7; t = 3.914; *P* = 0.001), those infected with *Giardia* (22.1 kg; 95% CI = 21.0, 23.2; t = 2.993; *P* = 0.003) and those who live in large families (>7 members) (23.5 kg; 95% CI = 22.8, 24.2; t = 2.267; *P* = 0.024) when compared to their peers. The results of the stepwise multiple linear regression model (adjusted for age) showed that the weight of children was found to be significantly influenced by the low household monthly income (β = −1.713; 95% CI = −2.56, −0.87; *P*<0.001), and *Giardia* infection (β = −1.658; 95% CI = −2.64, −0.68; *P* = 0.001) (Table 5). The overall R^2^ value of the regression model indicated that 58.9% of the variation in the weight of these children was explained by these variables.

**Table 4:** Mean weight and height at baseline by demographic, socioeconomic and parasitic infections factors of Aboriginal children who participated in the study.

Factors	Weight (kg)		Height (cm)	
	Mean (95% CI)	Statistics	Mean (95% CI)	Statistics
Age groups				
≥10 years	29.4(28.4, 30.4)	*t* = 16.162	134.9(133.7, 136.0)	*t* = 16.460
<10 years	21.0(20.5, 21.5)	*P*<0.001*	122.2(121.3, 123.1)	*P*<0.001*
Gender				
Female	24.3(23.3, 25.3)	*t* = 1.300	127.5(126.7, 129.0)	*t* = 1.985
Male	23.5(22.7, 24.2)	*P* = 0.188	125.6(124.2, 126.6)	*P* = 0.048*
Household monthly income				
≥MYR500	25.5(24.4, 26.6)	*t* = 3.914	128.3(126.8, 130.2)	*t* = 2.755
<MYR500 (low)	22.9(22.2, 23.7)	*P* = 0.001*	125.6(124.5, 126.6)	*P* = 0.006*
Fathers' education				
≥6 years formal education	24.4(23.4, 25.3)	*t* = 1.492	127.2(125.8, 128.6)	*t* = 1.349
No formal education	23.4(22.6, 24.2)	*P* = 0.136	125.9(124.7, 127.1)	*P* = 0.178
Mothers' education				
≥6 years formal education	25.1(24.0, 26.0)	*t* = 3.336	128.6(127.1, 130.0)	*t* = 3.992
No formal education	22.9(22.1, 23.7)	*P* = 0.001*	124.9(123.7, 126.0)	*P*<0.001*
Fathers' employment status				
Not working	24.1(23.4, 24.8)	*t* = −0.977	127.0(125.9, 128.1)	*t* = −1.407
Working	23.4(22.2, 24.7)	*P* = 0.329	125.6(123.7, 127.5)	*P* = 0.160
Mothers' employment status				
Not working	23.8(23.1, 24.6)	*t* = −0.304	126.5(125.4, 127.5)	*t* = 0.376
Working	24.2(22.8, 25.4)	*P* = 0.761	127.0 (124.9, 125.0)	*P* = 0.707
Family size				
≤7 members	25.2(24.2, 26.6)	*t* = 2.267	128.7(127.2, 130.6)	*t* = 2.501
>7 members (large)	23.5(22.8, 24.1)	*P* = 0.024*	125.9(124.8, 127.0)	*P* = 0.013*
*Giardia* infection				
Non infected	24.4(23.7, 25.2)	*t* = 2.993	127.0(125.9, 128.1)	*t* = 1.896
Infected	22.1(21.0, 23.2)	*P* = 0.003*	124.9(122.9, 126.9)	*P* = 0.059
*Ascaris* infection				
Negative to light infection	24.2(22.8, 25.9)	*t* = −0.854	126.9(125.2, 128.8)	*t* = −1.153
Moderate-to-heavy infection	25.5(24.2, 27.3)	*P* = 0.394	129.3(128.1, 130.7)	*P* = 0.250
*Trichuris* infection				
Negative to light infection	24.4(23.3, 26.3)	*t* = −0.096	126.9(124.5, 129.3)	*t* = −0.179
Moderate-to-heavy infection	24.5(23.5, 26.5)	*P* = 0.924	127.8(125.1, 129.7)	*P* = 0.858

MYR = Malaysian Ringgit; (US$1 = MYR3.05) [25^th^ February 2013].

* Significant difference (*P*<0.05; Independent samples t-test).

**Table 5:** Results of multiple linear regression of potential predictors for weight and height at baseline among Aboriginal schoolchildren who participated in the study *.

Variables	Weight				Height			
	β coefficient	Standard error	95% CI	*P*	β coefficient	Standard error	95% CI	*P*
Constant	3.241	1.148	-	-	81.743	1.454	-	-
Household monthly income (<MYR500)	−1.713	0.413	−2.56, −0.87	<0.001	-	-	-	-
*Giardia* infection (positive)	−1.658	0.499	−2.64, −0.68	0.001	-	-	-	-
Sex (male)	-	-	-	-	−2.677	0.544	−3.75, −1.61	<0.001
Mothers' educational level (<6 years)	-	-	-	-	−1.473	0.549	−2.55, −0.39	0.008

* Variables included in the multiple linear regression models were sex (male and female), household monthly income (<RM500 and ≥RM500), family size (≤7 members and >7 members), *Giardia* infection (infected and non infected) and mothers' educational level (≥6 years and <6 years).

On the other hand, the mean height was found to be significantly lower among those whose mothers had <6 years of education (124.8 cm; 95% CI = 123.7, 126.0; t = 3.992; *P*<0.001), those from families with a household monthly income of <RM500 (125.6 cm; 95% CI = 124.5, 126.6; t = 2.755; *P* = 0.006), and those who live in large families (125.9 cm; 95% CI = 124.8, 127.0; t = 2.501; *P* = 0.013) compared to their counterparts (Table 4). The output of the stepwise multiple linear regression model showed that the height of children was significantly predicted by the sex of children (male) (β = −2.677; 95% CI = −3.75, −1.61; *P*<0.001), and the low educational level of mothers (β = −1.473; 95% CI = −2.55, −0.39; *P* = 0.008) (Table 5). The overall R^2^ value of the regression model indicated that 68.3% of the variation in the height of these children was explained by these variables.

### Effects of *Giardia* infection on nutritional status

**Figure 3:**
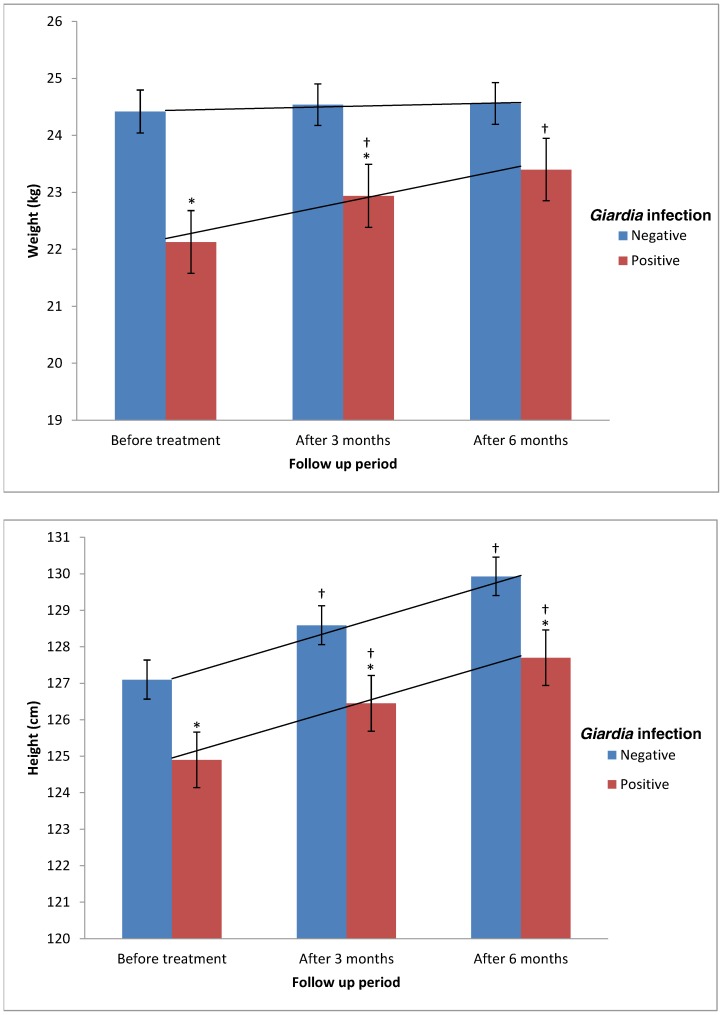
Mean weight and height of children according to *Giardia* infection over time. All values are mean (SEM). ^*^ Significant difference (lower) compared to non infected group (Independent t-test, *P*<0.05). **^†^** Significant difference (higher) compared to previous assessment (Paired t-test, *P*<0.05). Trend lines represent the linear weight and height increments (repeated measures ANOVA).

The mean values of weight and height were compared according to *Giardia* infection status over time and the results are presented in Figure 3. The mean weight of *Giardia*-infected participants was significantly lower than the mean weight of non infected participants (22.1 kg, 95% CI = 21.0, 23.2 vs 24.4 kg, 95% CI = 23.7, 25.2; t = 2.993; *P* = 0.003). Although the mean height of *Giardia*-infected participants was lower than the mean height of those non infected (124.9 cm, 95% CI = 122.9, 126.9 vs 127.1 cm, 95% CI = 125.9, 128.1), this difference was statistically not significant (t = 1.896; *P* = 0.059).

Three months after treatment for *Giardia* infection, a significant improvement in the mean weight of treated children (from 22.1 kg to 22.9 kg; paired t = 15.769; *P*<0.001) was observed. This significant improvement continued at 6 months assessment with mean increment of 0.5 kg (paired t = 5.861; *P*<0.001). Results of repeated-measures ANOVA confirmed the significant effect for time among *Giardia*-infected children, Wilks' Lambda = 0.161, F = 171.045, *P*<0.001 and multivariate eta squared = 0.676 (0.01 = small effect size, 0.06 = moderate effect, 0.14 = large effect). On the other hand, the improvement in weight among non infected children was found to be not significant at 3 months (from 24.4 kg to 24.5 kg; paired t = 1.788; *P*<0.075) and 6 months assessments (paired t = 1.590; *P* = 0.113). With regards to height, there were significant increments in the mean height of both groups (infected and non infected participants) after 3 and 6 months of treatment (*P*<0.001).

On the other hand, by adjusting the mean weight and height increments for age and sex (Table 6), the weight gain was found to be significantly higher among *Giardia*-infected children when compared to non infected children (*P*<0.05), regardless of age and sex. There was no significant difference in the mean height gain between both groups (*Giardia*-infected and non infected children) among those aged <10 years and male children. However, in children aged ≥10 years, height gain was significantly higher among *Giardia*-infected children when compared to those not infected. Similar results were reported among female children.

**Table 6:** Adjusted mean weight and height increments of children according to *Giardia* infection.

Group	*Giardia* infection	Weight			Height		
		0–3 months	3–6 months	0–6 months	0–3 months	3–6 months	0–6 months
Age group (years)							
≥10	Positive	0.9(0.6, 1.1)*	0.5(0.3, 0.7)*	1.3(1.0, 1.6)*	1.7(1.5, 1.9)	1.3(1.2,1.5)*	3.0(2.9, 3.3)*
	Negative	0.2(0.1, 0.3)	0.1(0.05, 0.2)	0.3(0.1, 0.5)	1.5(1.3, 1.7)	1.1(0.9, 1.1)	2.6(2.4, 2.8)
<10	Positive	0.8(0.6, 0.9)*	0.5(0.3, 0.7)*	1.3(1.0, 1.5)*	1.5(1.3, 1.7)	1.4(1.3, 1.7)	2.9(2.5, 3.3)
	Negative	0.2(0.1, 0.3)	0.1(0.07, 0.2)	0.3(0.1, 0.4)	1.5(1.3, 1.7)	1.4(1.2, 1.6)	2.9(2.6, 3.3)
Sex							
Male	Positive	0.9(0.7, 1.1)*	0.4(0.2, 0.6)*	1.3(1.0, 1.5)*	1.5(1.3, 1.7)	1.4(1.2, 1.7)	2.9(2.6, 3.2)
	Negative	0.1(0.05, 0.2)	0.1(0.05, 0.2)	0.2(0.1, 0.4)	1.5(1.3, 1.7)	1.5(1.3, 1.8)	3.0(2.7, 3.4)
Female	Positive	0.8(0.6, 0.9)*	0.5(0.3, 0.8)*	1.3(1.1, 1.6)*	1.6(1.3, 1.8)	1.3(1.2, 1.5)*	2.9(2.7, 3.3)*
	Negative	0.1(0.05, 0.2)	0.1(0.05, 0.2)	0.2(0.1, 0.3)	1.4(1.2, 1.6)	1.1(0.9, 1.2)	2.5(2.3, 2.7)
Overall	Positive	0.8(0.7, 0.9)*	0.5(0.3, 0.6)*	1.3(1.1, 1.4)*	1.6(1.4, 1.7)	1.3(1.1, 1.5)	2.9(2.7, 3.3)
	Negative	0.1(0.05, 0.2)	0.1(0.05, 0.2)	0.2(0.1, 0.3)	1.5(1.3, 1.6)	1.3(1.1, 1.5)	2.8(2.5, 3.0)

All values are mean (95% confidence interval).

* Significant difference compared to *Giardia*-negative children (*P*<0.05; Independent samples t-test).

Tables 7 and 8 show the mean changes of weight and height-for-age Z-scores adjusted for age and sex. Overall, significant improvements in the WAZ were reported in children treated for *Giardia* infection compared to those non infected, with significantly higher changes among those aged less than 10 years compared to their counterparts (*P*<0.05). Similar significant improvements in the WHZ were also reported. 

**Table 7:** Age-adjusted mean changes in weight and height-for-age Z-scores among children according to *Giardia* infection.

Variable	Age group (years)				Overall	
	≥10 years		<10 years			
	*Giardia* +ve	*Giardia* −ve	*Giardia* +ve	*Giardia* −ve	*Giardia* +ve	*Giardia* −ve
Δ WAZ (0–3 mo)	0.20(0.07,0.35)	0.14(0.06,0.22)	0.26(0.14,0.38)*	0.09(0.03,0.13)	0.23(0.14,0.33)*	0.11(0.06,0.15)
Δ HAZ (0–3 mo)	0.11(0.02,0.24)	0.08(0.01,0.18)	0.13(0.02,0.32)	0.05(0.01,0.13)	0.12(0.04,0.27)	0.06(0.01,0.16)
Δ WHZ (0–3 mo)	0.34(0.15,0.48)*	0.22(0.13,0.36)	0.26(0.12,0.33)	0.14(0.01,0.24)	0.30(0.22,0.50)*	0.17(0.13,0.25)
Δ WAZ (3–6 mo)	0.21(0.14,0.30)	0.18(0.11,0.24)	0.25(0.20,0.33)*	0.15(0.11,0.19)	0.23(0.19,0.30)*	0.16(0.12,0.19)
Δ HAZ (3–6 mo)	0.09(−0.01,0.25)	0.16(0.05,0.26)	0.15(0.05,0.26)	0.15(0.10,0.20)	0.12(0.04,0.21)	0.15(0.12,0.21)
Δ WHZ (3–6 mo)	0.21(0.08,0.44)	0.17(−0.06,0.26)	0.21(0.14,0.32)*	0.10(0.02,0.13)	0.21(0.15,0.32)	0.13(0.07,0.19)
Δ WAZ (0–6 mo)	0.40(0.31,0.57)	0.33(0.24,0.43)	0.49(0.34,0.59)*	0.24(0.19,0.32)	0.46(0.35,0.54)*	0.27(0.22,0.33)
Δ HAZ (0–6 mo)	0.20(0.10,0.38)	0.23(0.11,0.29)	0.30(0.16,0.54)	0.19(0.10,0.26)	0.26(0.12,0.44)	0.21(0.13,0.26)
Δ WHZ (0–6 mo)	0.50(0.41,0.71)*	0.28(0.16,0.39)	0.45(0.32,0.63)*	0.22(0.14,0.29)	0.48(0.36,0.62)*	0.27(0.16,0.35)

All values are mean (95% confidence interval); Δ: change; mo: months; WAZ: weight-for-age Z-scores; HAZ: height-for-age Z-scores; WHZ: weight-for- height Z-scores.

* Significant difference compared to *Giardia*-negative children (*P*<0.05; Independent samples t-test).

**Table 8:** Sex-adjusted mean changes in weight and height-for-age Z-scores among children according to *Giardia* infection.

Variable	Sex			
	Male		Female	
	*Giardia* +ve	*Giardia* −ve	*Giardia* +ve	*Giardia* −ve
Δ WAZ (0–3 mo)	0.23(0.15,0.35)*	0.09(0.03,0.14)	0.24(0.12,0.40)	0.12(0.05,0.19)
Δ HAZ (0–3 mo)	0.05(0.01,0.24)	0.06(0.01,0.12)	0.09(0.01,0.27)	0.05(0.01,0.12)
Δ WHZ (0–3 mo)	0.38(0.13,0.63)	0.20(0.11,0.29)	0.29(0.10,0.47)	0.18(0.09,0.27)
Δ WAZ (3–6 mo)	0.20(0.12,0.28)	0.16(0.11,0.22)	0.29(0.20,0.37)*	0.15(0.10,0.19)
Δ HAZ (3–6 mo)	0.18(0.01,0.22)	0.17(0.10,0.23)	0.16(0.02,0.30)	0.15(0.09,0.22)
Δ WHZ (3–6 mo)	0.22(0.09,0.39)	0.17(0.10,0.24)	0.25(0.14,0.35)	0.13(0.06,0.19)
Δ WAZ (0–6 mo)	0.45(0.34,0.57)*	0.26(0.18,0.33)	0.51 (0.38,0.59)*	0.27(0.22,0.36)
Δ HAZ (0–6 mo)	0.24(0.05,0.43)	0.22(0.10,0.29)	0.31(0.05,0.61)	0.19(0.10,0.27)
Δ WHZ (0–6 mo)	0.48(0.16,0.66)*	0.16(0.08,0.26)	0.36(0.19,0.55)	0.22(0.10, 0.33)

All values are mean (95% confidence interval); Δ: change; mo: months; WAZ: weight-for-age Z-scores; HAZ: height-for-age Z-scores; WHZ: weight-for- height Z-scores.

* Significant difference compared to *Giardia*-negative children (*P*<0.05; Independent samples t-test).

## Discussion

Parasitic diseases and malnutrition have a strikingly similar geographical distribution with the same people experiencing both insults together for much of their lives [35]. Our findings showed that the prevalence of *Giardia* infection among Aboriginal primary schoolchildren living in rural Malaysia was high with 22.0% of the participants infected. This prevalence is in agreement with other reports in Malaysia [5], [21], [36], [37]. The present study found that the overall prevalence of severe underweight, stunting and wasting was 26.9%, 24.8% and 21.0%, respectively. These high prevalence were in agreement with previous studies among Aboriginal children in different states [22], [38], [39].

The findings of the present study showed that age, low household monthly income and *Giardia* infection were the significant determinants influencing the weight of Aboriginal children. Similarly, the height of children was found to be significantly influenced by the age of children, sex (male) and the low educational level of mothers. Aside from age, which would be expected to affect stature, mothers' educational attainment and sex influenced height; paradoxically, females were taller than males in both age groups. From the results of linear regression models, it can be predicted that holding other variables unchanged, the weight of a child infected with *Giardia* is lower on average by 1.7 kg than non infected child. Previous studies have identified *Giardia* as a significant predictor of weight and height [17], [21], [40]. Moreover, *Giardia* infection has been identified as a significant risk factor of vitamin A deficiency among Aboriginal children in rural Malaysia [9].

It is well known that poverty is the root cause of malnutrition and many other health problems including parasitic infections in developing communities. Poverty limits the purchasing power of families and therefore, either the quantity or quality of food or may be both are compromised in these families. Education is also an important factor that contributes to the selection of the good quality and nutritious food. The low household monthly income and the low educational level of mothers have been identified as significant risk factors of malnutrition by previous studies from Malaysia [22], [23], [41]. Moreover, previous studies from Lao PDR, China and Bangladesh have reported similar findings [42], [43], [44]. Similarly, we found that children belonging to big families are more prone to be malnourished. This is common in rural and poor socioeconomic communities due to the inadequate purchasing power and also the distribution of food among family members.

Our findings showed that the mean weight and height of children were significantly lower among *Giardia*-infected children. This is consistent with previous findings of few reports in Malaysia and abroad. A previous study among Aboriginal children aged below 15 years reported that *Giardia* infection was a significantly associated with severe wasting (weight-for-height Z scores), but not with severe stunting (height-for-age) or severe underweight (weight-for-age) [21].

In comparison with studies from different countries, previous reports from Zimbabwe, Iran and Colombia found a strong association between *Giardia* infection and under-nutrition, wasting and stunting among children [45]–[47]. Previous studies among Brazilian children showed that *Giardia*-infected children had a double risk for stunted growth as compared to other children [48]. In a large, population-based survey of schoolchildren in Tehran, Nematian et al. [49] showed that among nine parasite species detected among the participants only *Giardia* and *Enterobius* infections were found to be significantly associated with the weight and height. In contrast, many studies have examined the association between *Giardia* infection and malnutrition and found no significant association [18]–[20]. This could be attributed to the low prevalence of *G. duodenalis* reported by these studies as compared to the present study.

Parasitic infections are thought to contribute to child malnutrition through subtle reduction in digestion and absorption, chronic inflammation and loss of nutrients [50]. *Giardia* is known to cause acute diarrhoea, fat, vitamins and D-xylose malabsorption, and lactose intolerance especially among children [8]. Moreover, it is well documented that *Giardia* trophozoites cause derangement of the normal villous architecture with shortening of villi and inflammatory foci in the crypts and lamina propria, resulting in malabsorption [50], [51]. All these mechanisms contribute to the development of malnutrition among infected individuals.

The findings of the present study showed a significantly higher post treatment weight gain among children who were reported to be infected with *Giardia* at baseline assessment as compared to those who were not infected. With regards to height, our findings showed significant post treatment height gain among all children throughout the follow up assessments, regardless of the *Giardia* infection status. However, the height gain among *Giardia*-infected females and those aged ≥10 years was significantly higher when compared to their non infected counterparts. This extra height gain among female and elder children could be due to physiological variation. Therefore, these findings confirmed the significant association of *Giardia* with weight of children but not with the height. Post treatment changes in the weight and height-for-age Z scores were found to be significantly higher among *Giardia*-infected children compared to those non infected, particularly among children aged below 10 years. This could be explained by their significantly higher prevalence of *Giardia* infection at baseline compared to their counterparts. Although more than 70% of the children studied were found to be infected with at least one species of soil-transmitted helminths (*Trichuris*, *Ascaris* and hookworm), a previous study showed no significant improvement in the weight and height of infected children after three months of complete deworming [52].

Evaluating the effects of *Giardia* on the nutritional status of children after treatment has been carried out by few studies and yielded different findings. In agreement with the findings of the present study, previous clinical trials reported that *Giardia*-infected children who have been successfully treated with metronidazole showed significantly higher improvement in weight and height as compared with those untreated [53], [54]. By contrast, Rousham and Mascie-Taylor [55] showed that after successful elimination of infection, *Giardia* was still found to be associated with poorer weight gain in children as compared to non treated children. In a randomized double placebo-controlled trial, Goto et al. [16] examined the relationship between *Giardia* and growth indicators (weight and height) of young children for 36 weeks and found no significant differences in the anthropometric variables between the intervention groups, although there were associations between improvement in small intestinal mucosal function and better weight-for-age and weight-for-height Z-scores. This study considered two approaches to define *Giardia* infection; the *Giardia* antibody titre contrasted and stool examination. However, the prevalence of *Giardia* was between 1% and 3% throughout the study period and this very low prevalence may mask any possible difference in growth improvement between the groups [16].

Although there is a broad agreement on the negative impacts of *Giardia* infection, a recent study showed that *Giardia* infection is associated with protection against diarrhea and fever without localizing signs [56]. A recent meta-analysis supported this finding and suggested that the presence of *Giardia* infection reduce the likelihood of having acute diarrhea but not the persistent diarrhea among children from developing countries [57]. Although the mechanism involved in this protection is still unclear, it was suggested that the secretion of innate antimicrobial products having anti-*Giardia* activity (eg, defensin) by the intestinal epithelium and the secretion of mucins and glycoproteins of the intestinal mucus layer can reduce attachment of many other enteropathogens to the mucosal surface [58]. Moreover, a significant reduction in the severity of rotavirus gastroenteritis was reported among infants in the presence of *Giardia* coinfection [59].

The present findings should be treated with some caution as a double blind randomization and comparison of treatment group with a placebo group were not possible. Several threats to internal validity of the before and after study design might be raised such as the instrumentation/reporting threat, regression-to-the-mean threat, maturation threat and dropout threats. To minimize the potential threats to the present study, non infected children served as control group and intention-to-treat approach for data analysis was used. Moreover, this study had to rely on a single fecal sample at each assessment stage (baseline, 3 months and 6 months) because of limitation of resources, the cultural belief of the Aboriginal people and to avoid causing interruption in the schoolchildren's schooling. Thus, the prevalence rate is expected to be higher if three fecal samples were collected. However, we have applied standard procedures during fecal collection and examination to overcome this limitation. The detection methods employed- trichrome staining and formalin ether sedimentation- are sufficiently sensitive to detect low numbers of *Giardia* parasite in fecal samples [26]. Aboriginal communities in rural Malaysia share similar socioeconomic, environmental and health profiles. Thus, we may speculate that our findings can be generalized to other rural Aboriginal children in other states. However, these results may not be generalizable to the entire Malaysian rural population as other ethnic groups (Malay, Chinese and Indians) have a better socioeconomic and environmental situation. Further studies are required to confirm these speculations.

### Conclusion

The present study reveals a high prevalence of *Giardia* infection and malnutrition among Aboriginal schoolchildren in rural Malaysia. In addition to the age, household monthly income and *Giardia* infection appears to be a strong predictor of weight but not height of the children, and a significant association of *Giardia* with the weight of children was reported. Thus, effective control measures to reduce the prevalence of *Giardia* infection should be considered in public health strategies to improve the nutritional status and quality of life of children in Aboriginal and other rural communities.

## Supporting Information

Checklist S1 STROBE checklist.
(DOC)
