# Supplementary material for: Burden of Giardia duodenalis Infection and Its Adverse Effects on Growth of Schoolchildren in Rural Malaysia
Source: PLoS Negl Trop Dis. 2013 Oct 31;7(10):e2516. doi: 10.1371/journal.pntd.0002516 (PMC3814875; doi:10.1371/journal.pntd.0002516)
Supplement: Checklist S1 — STROBE checklist. (DOC) [file pntd.0002516.s001.doc]

STROBE Statement—checklist of items that should be included in reports of observational studies

|  | Item No | Recommendation |
| --- | --- | --- |
| **Title and abstract** | 1 | (*a*) Indicate the study’s design with a commonly used term in the title or the abstract  Indicated. (Abstract); before and after treatment assessments |
|  |  | (*b*) Provide in the abstract an informative and balanced summary of what was done and what was found  Provided (Methods/Findings). |
| Introduction | | |
| Background/rationale | 2 | Explain the scientific background and rationale for the investigation being reported  Provided (paragraphs 1-3). |
| Objectives | 3 | State specific objectives, including any prespecified hypotheses  Provided (paragraphs 3). |
| Methods | | |
| Study design | 4 | Present key elements of study design early in the paper  Provided (first sentence in the Study area section). |
| Setting | 5 | Describe the setting, locations, and relevant dates, including periods of recruitment, exposure, follow-up, and data collection  All provided (Sections: Ethical statement, Study area, Study population and Treatment). |
| Participants | 6 | (*a*) *Cohort study*—Give the eligibility criteria, and the sources and methods of selection of participants. Describe methods of follow-up  Provided (Study population); it was a universal sampling in which all students present during the visit were invited to participate.  *Case-control study*—Give the eligibility criteria, and the sources and methods of case ascertainment and control selection. Give the rationale for the choice of cases and controls  *Cross-sectional study*—Give the eligibility criteria, and the sources and methods of selection of participants |
|  |  | (*b*) *Cohort study*—For matched studies, give matching criteria and number of exposed and unexposed  *Case-control study*—For matched studies, give matching criteria and the number of controls per case |
| Variables | 7 | Clearly define all outcomes, exposures, predictors, potential confounders, and effect modifiers. Give diagnostic criteria, if applicable  Provided (Section: Statistical analysis). |
| Data sources/ measurement | 8* | For each variable of interest, give sources of data and details of methods of assessment (measurement). Describe comparability of assessment methods if there is more than one group  Provided (Materials and Methods; Sections: Questionnaire, Anthropometric measurements, fecal samples examination) |
| Bias | 9 | Describe any efforts to address potential sources of bias  **Section: Fecal samples examination:** duplicate analysis was performed on 93 (25%) randomly collected samples; the samples were coded only with numbers and the technicians at the diagnostic laboratory were blinded to the code.  **Section: Anthropometric measurements:** the scale was calibrated regularly and measurements were taken twice by different persons and the mean value was recorded.  **Section: Statistical analysis:** These comparisons were adjusted for age and sex. |
| Study size | 10 | Explain how the study size was arrived at  Provided (Section: Study population) |
| Quantitative variables | 11 | Explain how quantitative variables were handled in the analyses. If applicable, describe which groupings were chosen and why  Provided (Section: Statistical analysis). |
| Statistical methods | 12 | (*a*) Describe all statistical methods, including those used to control for confounding  Provided (Section: Statistical analysis). |
|  |  | (*b*) Describe any methods used to examine subgroups and interactions  Provided (Section: Statistical analysis), A repeated-measures ANOVA |
|  |  | (*c*) Explain how missing data were addressed  Section: Statistical analysis and Figure 2 |
|  |  | (*d*) *Cohort study*—If applicable, explain how loss to follow-up was addressed  Figure 2  *Case-control study*—If applicable, explain how matching of cases and controls was addressed  *Cross-sectional study*—If applicable, describe analytical methods taking account of sampling strategy |
|  |  | (*e*) Describe any sensitivity analyses |

Continued on next page

| Results | | |
| --- | --- | --- |
| Participants | 13* | (a) Report numbers of individuals at each stage of study—eg numbers potentially eligible, examined for eligibility, confirmed eligible, included in the study, completing follow-up, and analysed  Considered. |
|  |  | (b) Give reasons for non-participation at each stage  Provided (Section Results: General characteristics of study population) |
|  |  | (c) Consider use of a flow diagram  Considered and provided (Fig 2). |
| Descriptive data | 14* | (a) Give characteristics of study participants (eg demographic, clinical, social) and information on exposures and potential confounders  Considered (Section Result). |
|  |  | (b) Indicate number of participants with missing data for each variable of interest  NA (No missing data). |
|  |  | (c) *Cohort study*—Summarise follow-up time (eg, average and total amount)  Figure 2 |
| Outcome data | 15* | *Cohort study*—Report numbers of outcome events or summary measures over time  Considered (Section Results) |
|  |  | *Case-control study—*Report numbers in each exposure category, or summary measures of exposure |
|  |  | *Cross-sectional study—*Report numbers of outcome events or summary measures |
| Main results | 16 | (*a*) Give unadjusted estimates and, if applicable, confounder-adjusted estimates and their precision (eg, 95% confidence interval). Make clear which confounders were adjusted for and why they were included  Considered. |
|  |  | (*b*) Report category boundaries when continuous variables were categorized  Considered (Section Anthropometric measurements). Z scores. |
|  |  | (*c*) If relevant, consider translating estimates of relative risk into absolute risk for a meaningful time period  NA |
| Other analyses | 17 | Report other analyses done—eg analyses of subgroups and interactions, and sensitivity analyses  NA |
| Discussion | | |
| Key results | 18 | Summarise key results with reference to study objectives  Considered. |
| Limitations | 19 | Discuss limitations of the study, taking into account sources of potential bias or imprecision. Discuss both direction and magnitude of any potential bias  Considered. Last paragraph of discussion. |
| Interpretation | 20 | Give a cautious overall interpretation of results considering objectives, limitations, multiplicity of analyses, results from similar studies, and other relevant evidence  Considered. |
| Generalisability | 21 | Discuss the generalisability (external validity) of the study results  Considered. Last paragraph of discussion. |
| Other information | | |
| Funding | 22 | Give the source of funding and the role of the funders for the present study and, if applicable, for the original study on which the present article is base |

*Give information separately for cases and controls in case-control studies and, if applicable, for exposed and unexposed groups in cohort and cross-sectional studies.

**Note:** An Explanation and Elaboration article discusses each checklist item and gives methodological background and published examples of transparent reporting. The STROBE checklist is best used in conjunction with this article (freely available on the Web sites of PLoS Medicine at http://www.plosmedicine.org/, Annals of Internal Medicine at http://www.annals.org/, and Epidemiology at http://www.epidem.com/). Information on the STROBE Initiative is available at www.strobe-statement.org.
